# Machine learning-based models for the prediction of breast cancer recurrence risk

**DOI:** 10.1186/s12911-023-02377-z

**Published:** 2023-11-29

**Authors:** Duo Zuo, Lexin Yang, Yu Jin, Huan Qi, Yahui Liu, Li Ren

**Affiliations:** 1 Department of Clinical Laboratory, Tianjin Medical University Cancer Institute & Hospital, Tianjin, 300060 China; 2 National Clinical Research Center for Cancer, Tianjin, 300060 China; 3 Tianjin’s Clinical Research Center for Cancer, Tianjin, 300060 China; 4 Key Laboratory of Cancer Prevention and Therapy, Tianjin, 300060 China; 5 Key Laboratory of Breast Cancer Prevention and Therapy, Tianjin Medical University, Ministry of Education, Tianjin, 300060 China; 6 Tongji University Cancer Center, Shanghai Tenth People’s Hospital, School of Medicine, Tongji University, Shanghai, 200072 China; 7 China Mobile Group Tianjin Company Limited, Tianjin, 300308 China

**Keywords:** Breast cancer, Machine learning, Artificial intelligence, Disease recurrence, Prediction model

## Abstract

Breast cancer is the most common malignancy diagnosed in women worldwide. The prevalence and incidence of breast cancer is increasing every year; therefore, early diagnosis along with suitable relapse detection is an important strategy for prognosis improvement. This study aimed to compare different machine algorithms to select the best model for predicting breast cancer recurrence. The prediction model was developed by using eleven different machine learning (ML) algorithms, including logistic regression (LR), random forest (RF), support vector classification (SVC), extreme gradient boosting (XGBoost), gradient boosting decision tree (GBDT), decision tree, multilayer perceptron (MLP), linear discriminant analysis (LDA), adaptive boosting (AdaBoost), Gaussian naive Bayes (GaussianNB), and light gradient boosting machine (LightGBM), to predict breast cancer recurrence. The area under the curve (AUC), accuracy, sensitivity, specificity, positive predictive value (PPV), negative predictive value (NPV) and F1 score were used to evaluate the performance of the prognostic model. Based on performance, the optimal ML was selected, and feature importance was ranked by Shapley Additive Explanation (SHAP) values. Compared to the other 10 algorithms, the results showed that the AdaBoost algorithm had the best prediction performance for successfully predicting breast cancer recurrence and was adopted in the establishment of the prediction model. Moreover, CA125, CEA, Fbg, and tumor diameter were found to be the most important features in our dataset to predict breast cancer recurrence. More importantly, our study is the first to use the SHAP method to improve the interpretability of clinicians to predict the recurrence model of breast cancer based on the AdaBoost algorithm. The AdaBoost algorithm offers a clinical decision support model and successfully identifies the recurrence of breast cancer.

**Supplementary Information:**

The online version contains supplementary material available at 10.1186/s12911-023-02377-z.

## Background

Breast cancer (BC) is one of the most common malignancies among women worldwide and a leading cause of cancer-related death in women [1]. The incidence has increased with the introduction of mammography screening, and BC cases in China account for 12.2% of all newly diagnosed breast cancers and 9.6% of all deaths from BC worldwide [2]. International studies suggest that approximately 30% of women will develop recurrence after the primary treatment for BC [3]. Patients with HR + breast cancer are at risk of recurrent disease even multiple decades after primary diagnosis [4]. Triple-negative BC have a high risk of distant relapse in the first 3 to 5 years following diagnosis [5]. Hence, the development of models to predict BC recurrence is important to aid in diagnosis and monitoring.

Breast cancer is a histologic diagnosis made based on standardized pathologic criteria. It primarily falls into invasive ductal carcinoma (60-75% of patients), invasive lobular carcinoma (5-15% of patients), and some special type carcinomas, making up the remainder of patients [6]. In BC, some pathological characteristics, such as estrogen receptor (ER), progesterone receptor (PR), and human epidermal growth factor receptor 2 (HER2), are used to guide treatment decisions. Due to the complex causes of BC, control, early diagnosis and appropriate treatment are important strategies for improving prognosis [7]. Downregulation of endoplasmic reticulum signaling by endocrine drugs is the primary systemic treatment for ER-positive or PR-positive breast cancers. HER2 is overexpressed in approximately 20% of breast cancers and is associated with poor prognosis in the absence of systemic therapy [8]. Patients with HER2- overexpressing breast cancer benefit from HER2-targeted therapy, including anti-HER2 antibodies (such as trastuzumab and pertuzumab) and small-molecule tyrosine kinase inhibitors (such as lapatinib and neratinib) [9].

The diagnosis and monitoring of BC are the main aspects of BC therapy. The information derived from patient and primary tumor features, specifically tumor size, nodal status, tumor grade, and therapeutic modalities, has been used to build prognostic models such as PREDICT [10]. However, despite considerable efforts at the early detection of recurrent disease, evidence suggests that only a small number of recurrent cases are detected at the asymptomatic stage [11, 12]. Multidisciplinary research or data mining is necessary to help physicians predict BC recurrence.

Recently, as artificial intelligence (AI) and its application in clinical cancer research have made rapid developments, cancer prediction performance has reached new heights [13, 14]. Powerful AI techniques, especially machine learning (ML) and deep learning (DL), can extract clinical information from massive amounts of data to assist in proper clinical decision making [15, 16]. These AI techniques are noninvasive techniques to diagnose the disease without harming the patient. ML is considered an objective and reproducible method for integrating multiple quantitative variables to improve diagnostic accuracy [17]. In population studies, ML can be used to effectively characterize BC risk, predict outcomes, and identify biomarkers without a priori assumptions of causation [18–20]. In breast cancer recurrence models, most studies have established predictive models based on imaging and pathological parameters [21–24]. Is it possible to use the clinical information obtained from the electronic medical records and the results of routine laboratory indicators to develop and verify the model for predicting the recurrence of BC?

This study explored and validated eleven predictive algorithms using an ML approach based on the clinicopathological and laboratory routine index data of BC patients. Our aim was to use the clinical information easily collected in clinical practice to create a clinical decision support system to identify patients at risk of recurrent cancer and promote early intervention in these patients.

### Literature survey

Currently, AI techniques and statistical methods is increasingly used and developed in clinical oncology to diagnose cancers, predict patient outcomes, and inform treatment planning. In particular, rich imaging and molecular data have stimulated the application of ML and/or DL. Recently, Manoj Sharma et al. [25] proposed a comparative analysis of handcrafted features extraction approaches and DL frameworks for colon and lung cancer classification. A significant improvement in classifiers performance is observed with features extracted by deep convolutional neural networks (CNNs). The random forest (RF) classifier with DenseNet-121 extracted deep features can identify colon and lung cancer tissue with excellent results. Similarly, the authors proposed a hybrid approach for survival prediction of hepatocellular carcinoma with more accuracy and sensitivity [26]. The proposed RFGBEL model presented excellent performance in contrast to other proposed models, which achieved an accuracy of 93.92%, sensitivity of 94.73%, F-1 score of 0.93. Yala et al. [27]proposed a DL model was built to triage mammograms by setting a high-sensitivity prediction threshold so that nearly all predicted negative cases were truly negative.

Many state-of-art studies have been presented for prediction of breast cancer. Manoj Sharma et al. [28] used an ensemble model comprising three pretrained CNNs to make grading predictions for the Databiox dataset, which consists of histopathological images of invasive ductal carcinoma breast cancer diagnosed patients for this grade classification and achieved an accuracy of 94%. Dhahri et al. [29] suggested an ML-based approach in combination of Genetic Programming to distinguish between benign and malignant breast tumors using electronic health records of 569 patients collected from the Wisconsin Breast Cancer dataset. In an experiment with seven classifiers, the adaptive boosting (AdaBoost) classifier performed best, with a fair accuracy of 98.23%, making it suitable for early BC detection in controlled parametric setting. Whitney et al. [30] used both ML and DL algorithms to analyze routine H&E-stained images of early-stage ER + breast cancer patients to predict the corresponding Oncotype DX recurrence risk. Bremer et al. [31] developed a biologic signature named DCISionRT for the calculation of individual decision score (DS), which combined molecular markers and clinicopathological factors associated with recurrence or progression of ductal carcinoma in situ patients following breast-conserving surgery in a nonlinear model.

On the one hand, many studies used limited clinical information analyzed by traditional statistical methods, and on the other hand, many studies analyzed image and pathology data by ML. From the literature survey, we found a relatively limited number of studies that predicted BC recurrence solely from easily accessible clinical information and routine laboratory metrics combined with ML. This study utilizes the results of clinical information and routine laboratory indicators obtained from electronic medical records combined with a comparison of 11 proposed ML models for predicting BC recurrence and is expected to present a rational model to help clinicians and decision makers.

## Materials and methods

### Patients

From January 2011 to December 2018, 342 hospitalized women diagnosed with primary BC at the Tianjin Medical University Cancer Institute and Hospital (Tianjin, China) were enrolled. All patients had complete pathological and clinical laboratory test results. Data were collected retrospectively, including patient characteristics, laboratory results, tumor size, lymph node staging (based on the eighth edition of AJCC) and treatment strategies (Table 1 and Supplementary Table 1).

**Table 1:** Characteristics of patients with breast cancer

	All patients	DR	No DR	χ^*2*^	*P*
N(%)	342 (100%)	256 (74.9%)	86 (25.1%)		
Age (mean ± SD, range, years)	46.65 ± 10.00 (19–77)	45.80 ± 10.10 (19–77)	49.19 ± 9.30 (28–69)	-	0.006
≤ 46	169 (49.4%)	135 (39.5%)	34 (9.9%)		
> 46	173 (50.6%)	121 (35.4%)	52 (15.2%)		
Primary site				0.060	0.806
Left	183 (53.5%)	136 (39.8%)	47 (13.7%)		
Right	159 (46.5%)	120 (35.1%)	39 (11.4%)		
Menopause				1.243	0.265
No	216 (63.2%)	166 (48.5%)	50 (14.6%)		
Yes	126 (36.8%)	90 (26.3%)	36 (10.5%)		
Histological type				6.967	0.008
Ductal	218 (63.7%)	153 (44.7%)	65 (19.0%)		
Others	124(36.3%)	103 (30.1%)	21 (6.1%)		
Tumor size				49.839	0.000
≤ 2 cm	102 (29.8%)	51 (14.9%)	51 (14.9%)		
2–5 cm	151 (44.2%)	122 (35.7%)	29 (8.5%)		
≥ 5 cm	83 (24.3%)	77 (22.5%)	6 (1.8%)		
N stage				80.151	0.000
N0	109 (31.9%)	50 (14.6%)	59 (17.3%)		
N1	77 (22.5%)	59 (17.3%)	18 (5.3%)		
N2	63 (18.4%)	58 (17.0%)	5 (1.5%)		
N3	93 (27.2%)	89 (26.0%)	4 (1.2%)		
Grading				33.202	0.000^a^
G1	15 (4.4%)	12 (3.5%)	3 (0.9%)		
G2	143 (41.8%)	83 (24.3%)	60 (17.5%)		
G3	163 (47.7%)	151 (44.2%)	12 (3.5%)		
G4	19 (5.5%)	8 (2.3%)	11 (3.2%)		
Molecular subtype				40.258	0.000^b^
Luminal A	23 (6.7%)	7 (2.0%)	16 (4.7%)		
Luminal B HER2-neg	149 (43.6%)	111 (32.5%)	38 (11.1%)		
Luminal B HER2-pos	29 (8.5%)	25 (7.3%)	4 (1.2%)		
HER2-pos	66 (19.3%)	59 (17.3%)	7 (2.0%)		
Triple negative	62 (18.1%)	54 (15.8%)	8 (2.3%)		
Unknown	13 (3.8%)	-	13 (3.8%)		
Treatment strategy				27.071	0.000^c^
Breast conserving	20 (5.8%)	6 (1.8%)	14 (4.1%)		
Mastectomy	252 (73.7%)	195 (57.0%)	57 (16.7%)		
Chemotherapy	64 (18.7%)	55 (16.1%)	9 (2.6%)		
Unknown	6 (1.8%)	-	6 (1.8%)		

Data are presented as numbers (percentages). The *P* value represents the result of statistical significance testing with χ^2^ test (or by a two-tailed Student’s *t*-test for age) for comparison between patients with disease recurrence (DR) and without relapse (no DR)

^a^χ^2^ test performed on classified groups (G1/G2 vs. G3/G4)

^b^χ^2^ test performed on classified groups (Luminal A/ Luminal B HER2-neg/ Luminal B HER2-pos/ HER2-pos/ Triple negative)

^c^χ^2^ test performed on classified groups (breast conserving/ mastectomy/ chemotherapy). Lymph node staging (N stage) was based on the eighth edition of AJCC

**Fig. 1:**
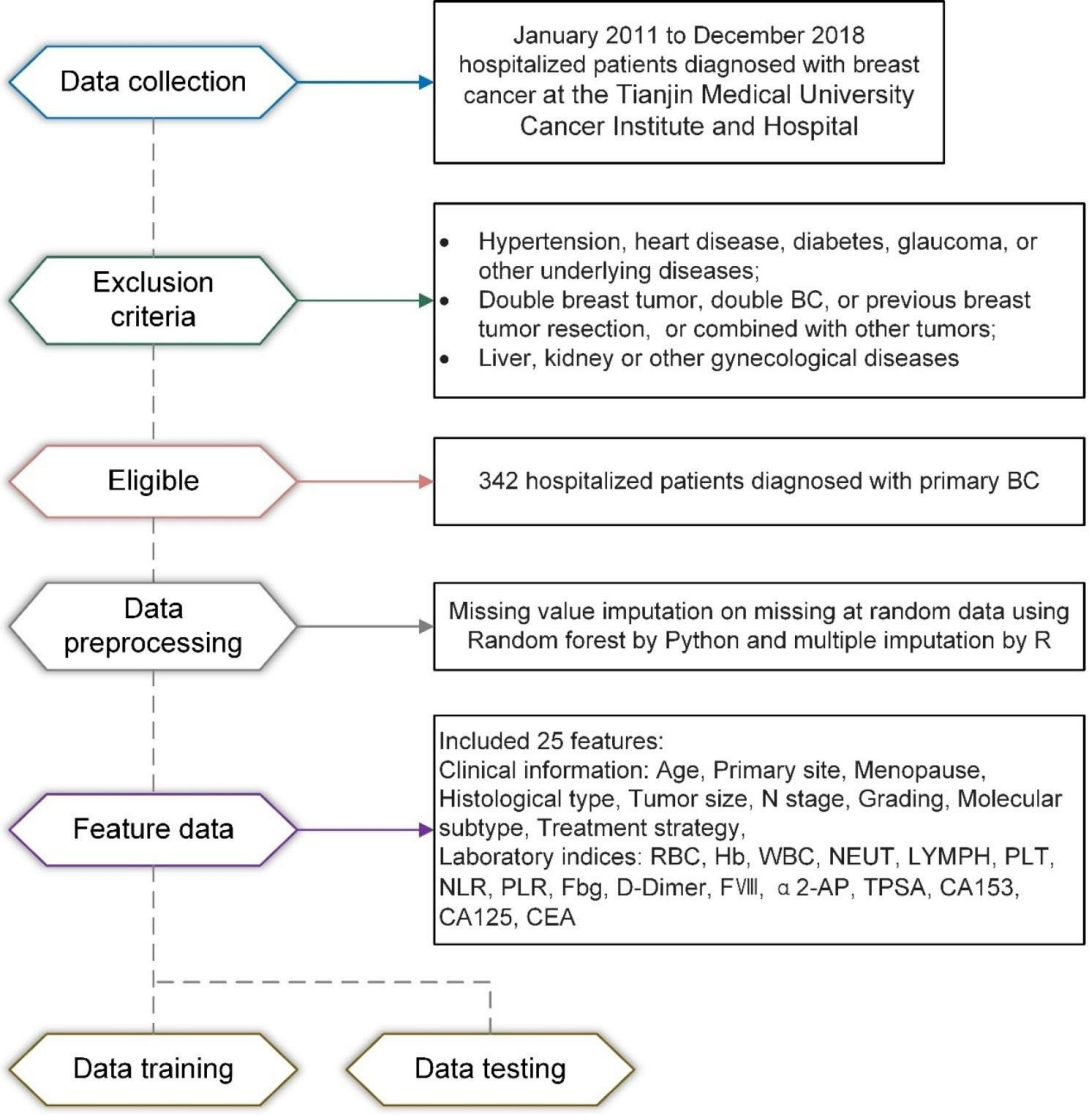
Visual diagram of the detailed process for clinical design and data collection

The inclusion criteria were as follows: (1) patients who met the diagnostic criteria for BC and were confirmed by pathological examination; (2) women with unilateral breast lesions for the first time; (3) patients who had not received chemotherapy, radiotherapy, or endocrine therapy; and (4) patients with complete clinical and pathological data. The exclusion criteria were as follows: (1) patients with hypertension, heart disease, diabetes, glaucoma, or other underlying diseases; (2) patients with double breast tumor, double BC, or previous breast tumor resection; (3) patients with other tumors; (4) patients with an intellectual disability or other serious mental illness; and (5) patients with liver, kidney, or other gynecological diseases. The process is shown in Fig. 1.

### Data preprocessing

For patient information, we converted “patients over 46 years old” to 1 and “patients ≤ 46 years old” to 0. For the diagnosis code, we converted “patients with BC recurrence” to 1 and “patients with no recurrence” to 0; We converted “patients with menopause” to 1 and “patients with no menopause” to 0; We converted “patients with primary cancer in the left breast” to 1 and “patients with primary cancer in the right breast” to 2; We converted “patients with invasive ductal carcinoma of BC” to 1, “patient with other types of invasive carcinoma” to 0, and “patients with unknown histological types of BC” to 2; We converted “patient with tumor size ≤ 2 cm” to 0, “patients with tumor size > 2 cm and ≤ 5 cm” to 1, “patients with tumor size > 5 cm” to 2, “patients with unknown tumor size” to 3; We converted “patient with lymph node staging 0” to 0, “patient with lymph node staging 1” to 1, “patient with lymph node staging 2” to 2, and “patient with lymph node staging 3” to 3; We converted “patient with unknown of histological grade” to 0, “patient with more well-differentiated histological grade” to 1, “patient with moderately differentiated histological grade” to 2, and “patient with more poorly differentiated histological grade” to 3 and “patient with undifferentiated histological grade” to 4; We converted “patient with HER2-positive of molecular subtype” to 1, “patient with Triple-Negative Breast Cancer (TNBC)” to 2, “patient with Luminal A of molecular subtype” to 3, “patient with Luminal B HER2- negative of molecular subtype” to 4, “patient with Luminal B HER2-positive of molecular subtype” to 5, and “patient with unknown of molecular subtype” to 6; We converted “patient with breast conserving therapy” to 0, “patient with mastectomy” to 1, “patient with chemotherapy” to 2, and “patient with unknown treatment strategies” to 3. Missing value imputations on missing at random data of laboratory indicators were used random forest by Python package (Sklearn, 1.0.2) and multiple imputation by R package (mice, 4.1.2).

### Machine learning models

**Fig. 2:**
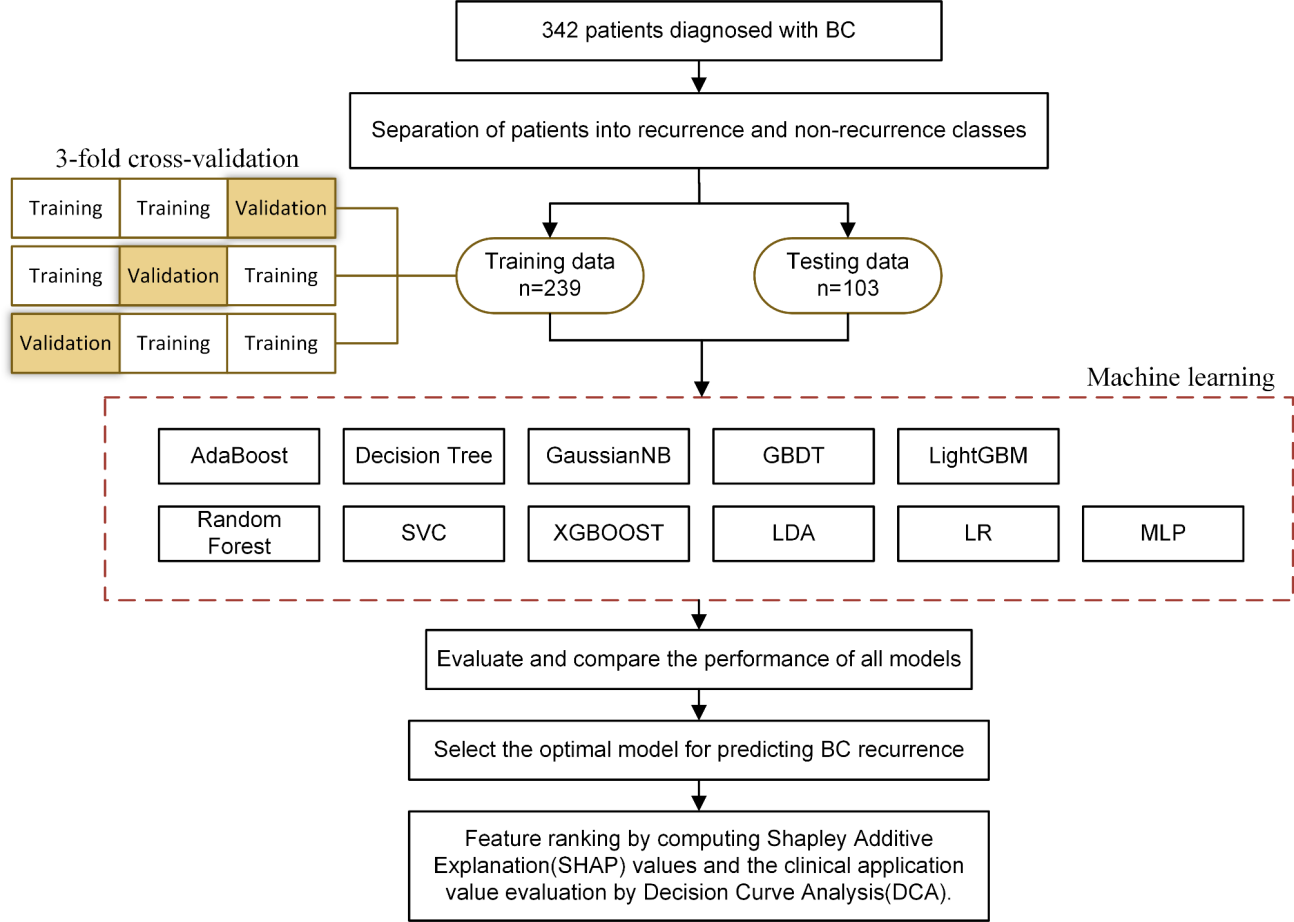
Flowchart of the machine learning development model for predicting recurrence of breast cancer. Abbreviations: BC, breast cancer; LR, logistic regression [32]; RF, random forest [33]; SVC, support vector classification [34]; XGBoost, extreme gradient boosting [35]; GBDT, gradient boosting decision tree [36]; decision tree [37]; MLP, multilayer perceptron [38]; LDA, linear discriminant analysis [39]; AdaBoost, adaptive boosting [40]; GaussianNB, Gaussian naive Bayes [41]; LightGBM, light gradient boosting machine [42]; SHAP, Shapley Additive Explanation; DCA, decision curve analysis

The prediction model was developed by using the following algorithms: logistic regression (LR) [32], random forest (RF) [33], support vector classification (SVC) [34], extreme gradient boosting (XGBoost) [35], gradient boosting decision tree (GBDT) [36], decision tree [37], multilayer perceptron (MLP) [38], linear discriminant analysis (LDA) [39], AdaBoost [40], Gaussian naive Bayes (GaussianNB) [41], and light gradient boosting machine (LightGBM) [42]. All ML analyses were performed by Python 3.8.8. The study samples were randomly divided into a training set (n = 239) and a testing set (n = 103) at a ratio of 7:3 [43, 44]. In the process of training, we used a 3-fold inner cross-validation approach to estimate the models [45, 46]. In the test set, the AUC, accuracy, sensitivity, specificity, positive predictive value (PPV), negative predictive value (NPV) and F1 score were estimated. The best prediction model was selected by evaluating the largest AUC [47, 48]. We applied the Shapley Additive Explanation (SHAP) to explain the best-performing predictive model. Feature ranking was obtained by computing SHAP values. The features were ordered by the mean absolute value of the SHAP values for each feature [44]. Combining ML with SHAP could provide an explicit explanation of the efficacy prediction [44, 47, 49]. The process is shown in Fig. 2.

### Statistical analyses

Categorical data were analyzed using the chi-square test [50]. If the two sets of continuous variables were normally distributed, a two-tailed Student’s *t*-test was used for comparison. If the two sets of continuous variables were nonnormally distributed, a Mann‒Whitney test was used for comparison. The cumulative risk curve was drawn by Kaplan‒Meier methods. The cumulative risk incidence between the two groups was compared based on Kaplan‒Meier analysis and the log-rank test. SHAP and decision curve analysis (DCA) were performed using Python 3.8.8. Statistical analysis was conducted by SPSS statistics 25.0. All statistical tests were two-tailed, and *p* < 0.05 was considered significant.

## Results

### Clinical features

In all, 342 BC patients (average age, 46.65 years; range, 19–77 years) from January 2011 to December 2018 were identified, and 256 (74.9%) had recurrence, 86 (25.1%) patients had no recurrence. Table 1 summarizes the clinical and tumor histological characteristics of patients. The most frequent molecular subtypes were luminal B HER2-neg (43.6%) > HER2-pos (19.3%) > TN (18.1%) > luminal B HER2-pos (8.5%) > luminal A (6.7%) [missing data for molecular subtypes were grouped as unknown (3.8%)]. Compared with patients without recurrence, patients with recurrence had multiple lymph node involvement and invasive ductal-type disease, and the histological grade and tumor size in these patients were significantly higher (Table 1). In clinical laboratory characteristics, D-dimer, CEA, CA125, CA15-3, WBC, NEUT, NLR, Fbg and α2-AP levels played critical roles in the differential diagnosis of patients with BC recurrence and no recurrence (Supplementary Table 1). All of these clinical features are easily obtainable from the electronic medical records of BC patients, and there are a total of 25 clinical features.

### Machine learning-based prediction of BC recurrence

**Fig. 3:**
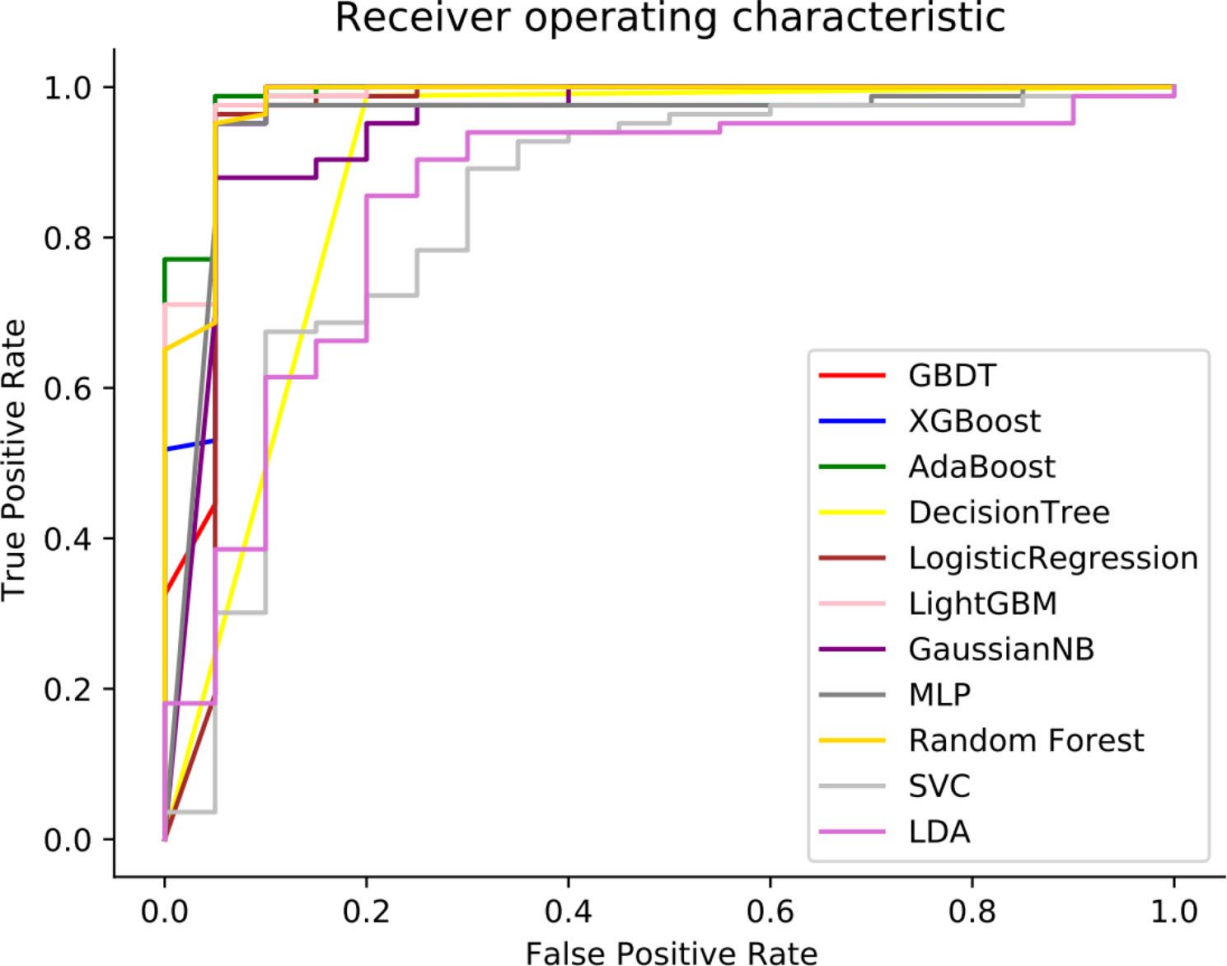
Comparison of the area under the receiver operating characteristic curves for eleven machine learning algorithms. Abbreviations: LR, logistic regression; RF, random forest; SVC, support vector classification; XGBoost, extreme gradient boosting; GBDT, gradient boosting decision tree; MLP, multilayer perceptron; LDA, linear discriminant analysis; AdaBoost, adaptive boosting; GaussianNB, Gaussian naive Bayes; LightGBM, light gradient boosting machine

We hypothesized that the comprehensive integration of clinical features might provide important clues to predict BC recurrence outcomes. Therefore, we obtained 25 clinical features from electronic medical records, all of which were used for the development of predictive models for BC recurrence. We tested the performance of eleven ML models, including AdaBoost, LightGBM, XGBoost, decision tree, GBDT, LDA, GaussianNB, SVC, LR, RF and MLP, using the discovery cohort. We selected and then tested eleven types of ML models as clinical decision-support systems for predicting BC recurrence. During the development of these models, the clinical features of 70% of the patients were randomly selected for training. In addition, we performed a 3-fold internal cross-validation used to assess the effectiveness of the predictive ability of a model built based on the training set, and externally validated the accuracy of the predictive ability of the model by going through a test set based on an independent sample size (Table 2 and Supplementary Table 2). Furthermore, to evaluate the performance of a ML model, a confusion matrix was used (Supplementary Table 3). The prediction performances of these eleven ML models were compared, and the most accurate prediction model was chosen. The model obtained by AdaBoost had the best discrimination (AUC = 0.987) (Fig. 3). The sensitivity, specificity, PPV, NPV, F1 score and accuracy of the model for predicting BC recurrence were 94.7%, 97.6%, 90.0%, 98.8%, 92.3% and 97.1%, respectively (Table 2).

**Table 2:** Comparison of the prediction results of each test model using test datasets

Algorithms	AUC	Accuracy		Sensitivity	Specificity	PPV	NPV	F1 Score
AdaBoost	0.987		0.971	0.947	0.976	0.900	0.988	0.923
Decision Tree	0.894		0.951	0.941	0.953	0.800	0.988	0.865
GaussianNB	0.945		0.883	0.667	0.949	0.800	0.904	0.727
GBDT	0.967		0.971	0.947	0.976	0.900	0.988	0.923
LightGBM	0.983		0.971	0.947	0.976	0.900	0.988	0.923
LR	0.951		0.961	0.864	0.988	0.950	0.964	0.905
MLP	0.952		0.951	0.857	0.976	0.900	0.964	0.878
Random Forest	0.981		0.981	1.000	0.976	0.900	1.000	0.947
SVC	0.834		0.864	0.750	0.879	0.450	0.964	0.563
XGBoost	0.974		0.971	0.947	0.976	0.900	0.988	0.923
LDA	0.847		0.883	0.722	0.918	0.650	0.940	0.684

Abbreviations: PPV, positive predictive value; NPV, negative predictive value; LR, logistic regression; RF, random forest; SVC, support vector classification; XGBoost, extreme gradient boosting; GBDT, gradient boosting decision tree; MLP, multilayer perceptron; LDA, linear discriminant analysis; AdaBoost, adaptive boosting; GaussianNB, Gaussian naive Bayes; LightGBM, light gradient boosting machine

**Fig. 4:**
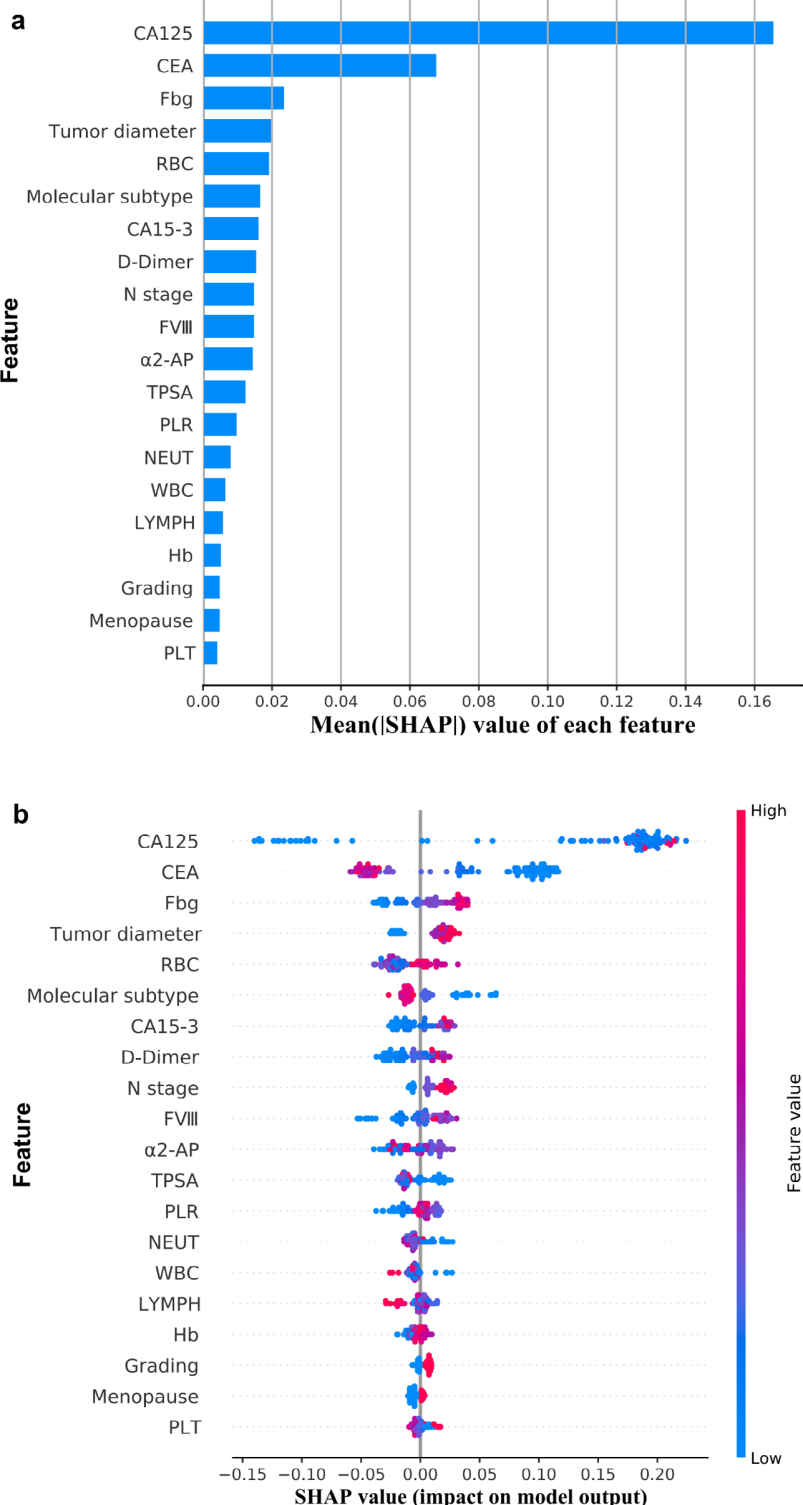
SHAP values and feature interaction scores in AdaBoost-based prediction. (**a**) The top 20 most important features for the prediction of BC recurrence (ranked from most to least important). (**b**) The distribution of the impacts of each feature on the model output. The colors represent the feature values: red for larger values and blue for smaller values. Abbreviations: CA125, carcinoma antigen 125; CEA: carcinoembryonic antigen; Fbg: fibrinogen; CA15-3, carcinoma antigen 15 − 3; FVIII, coagulation factor VIII; TPSA, tissue polypeptide-specific antigen; α2-AP, α2-antiplasmin; RBC, red blood cell; NEUT, neutrophils; PLR, platelet-to-lymphocyte ratio; WBC, white blood cell; PLT, platelet, SHAP, Shapley Additive Explanation

The AdaBoost algorithm was adopted in the establishment of the prediction model. To better understand how features in the prediction model of BC recurrence based on the AdaBoost algorithm contribute to the prediction results, we calculated the SHAP value of each feature. The top 20 features were selected by the importance ranking of the average absolute SHAP value, which was based on the AdaBoost algorithm model (Fig. 4a). According to the importance ranking of the average absolute SHAP value, the top 4 features [carcinoma antigen 125 (CA125), carcinoembryonic antigen (CEA), fibrinogen (Fbg) and tumor diameter] were assessed as the most important variables. Figure 4b is a violin plot of each feature showing the correlation between the value of each feature and the SHAP value. The larger the absolute value of a feature’s SHAP, the greater the hint that this feature has a greater impact on the AdaBoost-based prediction model. Red dots represent the higher values for this feature, while blue dots represent lower values for this feature. Higher CA125, Fbg, carcinoma antigen 15 − 3 (CA15-3), D-dimer and coagulation factor VIII (FVIII) concentrations, red blood cell (RBC) count, N stage, larger tumor diameter and lower CEA, α2-antiplasmin (α2-AP) and tissue polypeptide specific antigen (TPSA) concentrations were associated with a higher predicted probability of 5-year BC recurrence. Furthermore, different molecular subtypes also had a certain impact on the outcome of BC recurrence.

### Clinical use

**Fig. 5:**
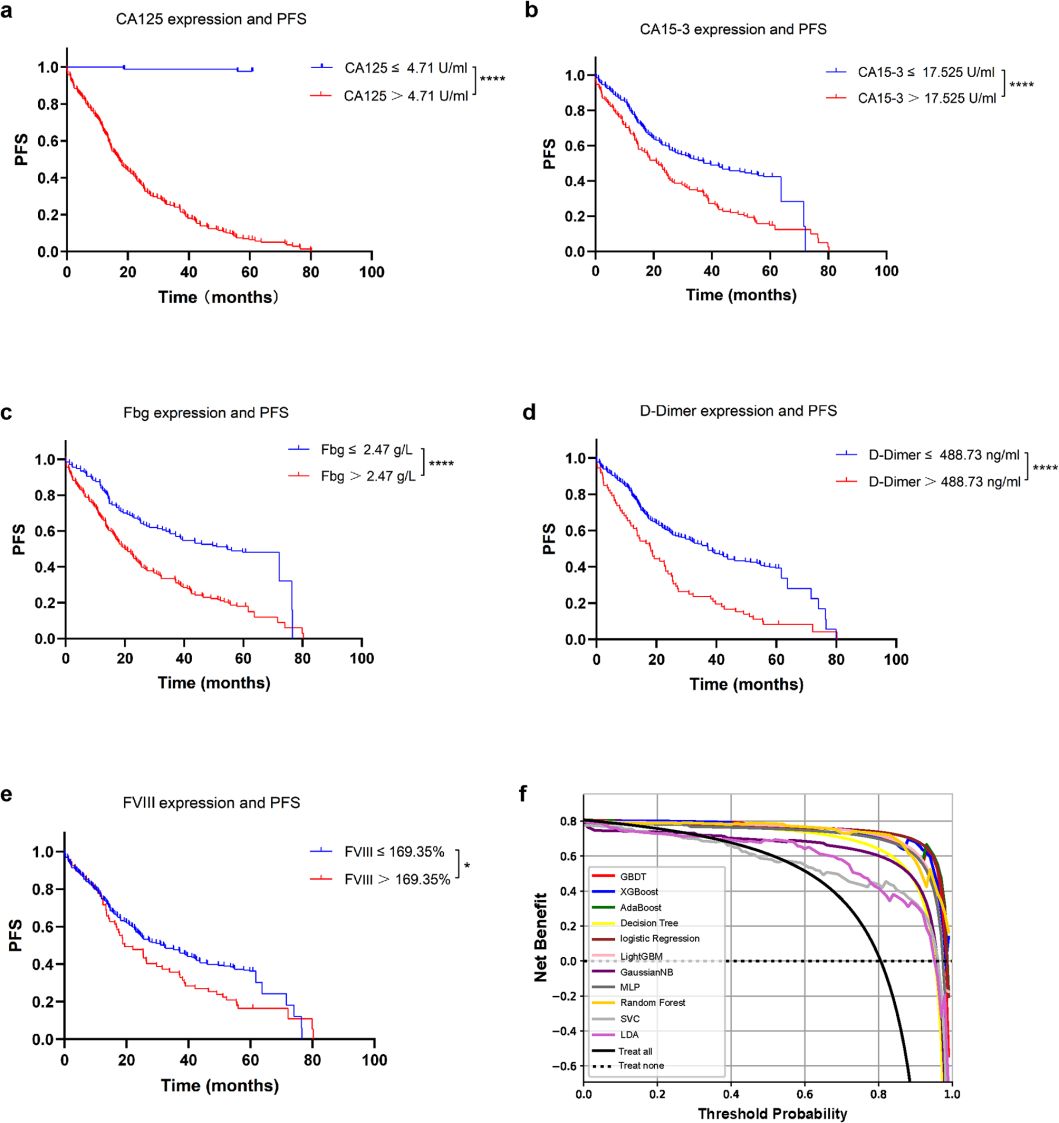
Kaplan‒Meier plots and decision curve analysis (DCA). (**a**–**e**) Kaplan‒Meier plot of progression-free survival (PFS) based on CA125, CA15-3, Fbg, D-Dimer and FVIII expression (*p < 0.05, ****p < 0.0001). (**f**) DCA of different ML algorithms. The y-axis measures the net benefit. The dotted line (Treat None) represents the net benefit of the prediction of nonrecurrence for all BC patients; the black line (Treat All) represents the net benefits of the outcomes of recurrence for all BC patients. The model with the highest clinical value was determined by quantifying net benefits under different thresholds

Several single clinical features were found to be significant predictive markers of BC recurrence. There were significant correlations between CA125 expression (cutoff 4.71 U/ml) and BC prediction. The cutoff value was selected based on the probability threshold of Youden’s index. The study population was divided into high-risk groups and low-risk groups based on the cutoff value. Based on Kaplan‒Meier analysis and the log-rank test, there was a significant difference in progression-free survival between the two groups (*p* < 0.0001, Fig. 5a). CA125 expression levels affected the risk of recurrence, with higher expression levels associated with a higher five-year risk of recurrence and a shorter progression-free survival in patients. Similarly, higher expression levels of CA15-3, Fbg, D-dimer and FVIII were correlated with a worse prognosis in patients (Fig. 5b–e).

In clinical practice, machine model prediction is not simply predictive of patients who will likely have BC recurrence or be free of recurrence. The clinical application value of the model was evaluated by DCA. We expressed the net benefit as a function of the decision threshold in the decision curve, and the threshold probability reflected the cost‒benefit ratio. The DCA of the 11 ML algorithms is shown in Fig. 5f, which shows that when the threshold probability of a patient was greater than 1%, using the AdaBoost algorithm model to guide clinical intervention provided more benefit than either the intervention for all (black line) or none (dotted line). Compared with the other algorithms, the net benefit in this range had obvious superiority. When 1% was taken as the prediction probability, the net benefit of the AdaBoost algorithm was significantly higher than that of the other algorithms.

## Discussion

In this study, an AdaBoost-based model was trained and tested as a decision-making tool, which is expected to predict the recurrence risk of BC. In addition, the most important variable features were selected by SHAP from many clinical characteristics related with BC. With this type of analysis, clinicians can use the model established by the proposed algorithm to identify BC patients with high recurrence risk, and it is expected to improve the risk stratification of patients in clinical practice.

BC, the most common malignancy diagnosed in women worldwide, is a highly heterogeneous disease presenting with a broad range of clinical and molecular characteristics. In recent years, there has been a steady decline in BC mortality, and early detection of BC recurrence allows for more effective salvage treatment. Women with early BC are at an ongoing risk of relapse, even after successful surgery and treatment of the primary tumor [51]. Following initial treatment, BC can recur locally, regionally (nodes), or at distant metastatic sites. For women with HR-negative disease, the risk of recurrence is mainly confined to the first 5 years after diagnosis [52, 53]. Patients with HR-positive tumors are at risk of late recurrence even after triamcinolone therapy [54]. Most recurrences of BC occur distally, with the most common sites of metastasis being the bones, liver and lungs [55]. Although BC tumor markers such as CA15-3 and CEA can be used to detect early tumor recurrence, the serum test is not suitable alone for BC follow-up [56, 57]. To establish a predictive model for BC recurrence with comprehensive integration of relevant clinical factors, we collected 25 clinically relevant features that are clinically common and readily available from patients with BC from electronic medical records, including CA125 expression, coagulation function (Fbg, FVIII and D-dimer), tumor diameter, molecular subtype, and previous strategy of therapy, among others. These factors are evaluated in routine clinical practice and do not require additional cost or effort.

CA125 is expressed by normal bronchial, endometrial, ovarian and corneal epithelial cells, and it was first identified in mice immunized with ovarian cancer cells [58]. CA125 is best known as a biomarker for monitoring epithelial ovarian cancer [59]. In addition, CA125 is a repeating peptide epitope of the mucin MUC16, which promotes breast cancer cell proliferation and metastasis [60, 61]. An increase in the concentration of CA125 is an indicator of disease recurrence [62]. In a study by Jager et al., CA 125 levels in 26 patients with a single site of metastasis out of 250 metastatic BC patients were analyzed, suggesting that an elevated CA 125 level in metastatic BC patients is related to pleural disease [63]. Another prospective study also demonstrated the value of metastatic sites. Among nine patients with pleural-based disease, eight (89%) had an elevated CA 125 level, and progressive disease correlated with elevated CA 125 levels in all cases [64]. This suggests that lesions close to the pleura can induce an inflammatory reaction and result in elevated CA 125 levels. In a retrospective review of 51 patients with treated BC, progressive disease in 21 patients correlated with an elevation in CA125 in 57% of patients and one false-negative reduction [65]. Continuous biomarker monitoring has the potential to predict the diagnosis of recurrence at the minimum asymptomatic stage [66]. Our data demonstrate that an increase in CA 125 may also result in the earlier detection of recurrent or progressive disease, which is likely to alter survival and affect quality of life. The patients with values above the cutoff of CA125 presented a significantly shorter median PFS than those with values below the cutoff. The optimal use of this marker in breast carcinomatosis is unclear, but its possible use in combination with other tumor markers, such as CA15-3 or CEA, is expected to be of complementary value for clinical decision making and to improve our understanding of the function of CA125 in human pathology.

CEA is a cytoplasmic glycoprotein that is highly expressed in most tumor tissues and is commonly used as a marker to assess cancer risk and prognosis [67, 68]. However, this study reveals conclusions that are inconsistent with these several studies, and low CEA expression levels are involved in predicting the recurrence risk of BC according to SHAP values. Similarly, in 105 patients with metastatic BC, 39 patients (37%) with low CEA levels had significantly shorter median survival times after recurrence (18 versus 28 months) than patients with high CEA levels [69]. Low CEA levels may indicate complex and heterogeneous disease; thus, there might be a subtype of BC with rapid proliferation and low CEA secretion [69]. Preoperative serum levels of CEA were associated with molecular subtypes of BC, and CEA expression levels were significantly lower in patients with triple-negative metastatic BC than in those with other subtypes [70].

Malignant tumor growth and dissemination are associated with the development of a subclinical hypercoagulable state [71]. The patient’s coagulation abnormalities worsened with cancer progression and metastasis. In this respect, circulating thrombotic biomarkers may represent a novel noninvasive factor for better prediction of disease recurrence risk [72]. In our study, fibrinogen, FVIII and D-dimer had potential value in predicting BC recurrence. D-dimer is the primary degradation product of cross-linked fibrin, representing an index of both coagulation and fibrinolysis activation. The pathogenesis of cancer coagulation activation is complex and variable. Laboratory results indicate that fibrinolysis and fibrinolysis processes are similar in the progression of malignant tumors and are increasingly present in patients with metastases [73]. In BC, high fibrinogen levels were associated with poorer overall survival [74, 75]. Some studies have shown that D-dimers are useful indicators for monitoring metastasis in cancer patients, and increased D-dimer levels are associated with the rate of progression and poor prognoses [76, 77], which is consistent with our data.

A growing body of evidence suggests that the risk of recurrence depends heavily on the biology of BC [78–80]. The classification of subtypes shows the heterogeneity of BC, which has been shown to be of prognostic value in BC. Several studies have revealed associations between molecular subtypes of BC and local recurrence rates. The subtypes are ER-positive luminal A (luminal A), ER-positive luminal B (luminal B), HER2 enriched, basal-like, and normal breast-like. In a study of 2985 patients classified into different subtypes, HER2-enriched and basal subtypes showed a significantly higher risk of regional relapse after breast-conserving therapy [81]. Luminal B tumors have poorer outcomes than luminal A tumors due to the expression of some proliferating genes, such as Ki-67, CCNB1 and MYBL2 [82]. In addition, tumor size and lymph node status were significant predictors of disease-free survival and overall survival. In a cohort of 15,819 women with invasive BC, the rate of lymph node metastasis increased with increasing tumor volume in BC patients with tumors smaller than 100 cm^3^, increasing BC mortality [83].

The strong heterogeneity of BC represents a serious issue for treatment monitoring [84], and predicting the individual risk of recurrence of primary BC will enable physicians to choose the best treatment strategy. In this respect, AI holds great promise to enable the evaluation of tumor aggressiveness, individual risk of recurrence, and response to specific treatments in BC [85]. AI is applied to assist cancer diagnosis and prognosis, given its unprecedented accuracy level, which is even higher than that of general statistical experts [14]. Previous studies have mainly applied AI to two main approaches to BC diagnosis, relying on image analysis and pathological data [86]. While AI in digital breast pathology and breast imaging shows great promise in reducing false positive rates in breast cancer screening, images might suffer from technical bias [86, 87]. In this study, we used clinical characteristics, pathological molecular typing, and laboratory indicators, which provide a detailed fingerprint of tumors to predict recurrent BC by ML-based AI.

ML, as a narrow form of AI, has been proven to be a powerful tool in the prediction of disease outcomes [88–92]. In our study, prediction models based on 11 ML algorithms were tested using 25 easily obtainable clinical features from electronic medical records. Compared with the prediction performance of every single clinical feature, ML-based AdaBoost using the combination of clinical features showed more significant performance. Several recent studies have used ML methods to predict cancer recurrence and survival outcomes. For example, a study showed three prediction models combined with digitized images of fine needle aspiration of breast masses that can be used to predict BC reoccurrence time as accurately as 1 year [93]. In addition, Tahmassebi A reported using ML with multiparametric magnetic resonance imaging to predict pathological complete response and survival in patients treated with neoadjuvant chemotherapy [94]. A breast cancer recurrence and metastasis risk assessment framework was developed from histopathological images using image features and ML technologies [23]. In contrast to these studies, we tested more models based on different algorithms for predicting BC recurrence within a five-year follow-up period through easily accessible clinical information and routine laboratory indicators. We found that AdaBoost can be used to predict recurrence/nonrecurrence with an accuracy of 97.1%, a high sensitivity of 94.7% and a high specificity of 97.6%.

To our knowledge, we used AdaBoost in combination with SHAP for the first time to predict the recurrence of BC. Second, by searching for keywords [(((((((conventional laboratory indicators) OR (routinely measured blood biomarkers)) OR (routinely measured blood indicators)) OR (routinely peripheral blood indicators)) OR (conventional peripheral blood indicators)) AND ((breast cancer) OR (breast carcinoma))) AND (Recurrence)) AND ((((((((((((Machine Learning) OR (logistic regression)) OR (random forest)) OR (support vector machine)) OR (XGBoost)) OR (gradient boosting decision tree)) OR (decision tree)) OR (multilayer perceptron)) OR (linear discriminant analysis)) OR (AdaBoost)) OR (Gaussian naive Bayes)) OR (LightGBM))] on the PubMed website, we believe that our study is the first to use the features of traditional laboratory indicators and clinical information easily available from electronic medical records in AdaBoost’s model to predict the recurrence of BC. AdaBoost is one of the best boosting algorithms. AdaBoost can boost a weak learning algorithm with an accuracy slightly better than random guessing into an arbitrarily accurate strong learning algorithm, bringing about a new method and new insights into the design of the learning algorithm [95]. Even if many base classifier instances are used, AdaBoost rarely overfits the solution and minimizes the exponential loss function by fitting the stepwise additive model [96]. Due to the minimization of the classification error, which can be best approximated as exponential loss, AdaBoost performs very well on a wide range of classification problems [97] AdaBoost could be a helpful tool for physicians to predict BC recurrence. Additionally, we use SHAP to interpret AdaBoost predictions based on SHAP values and feature interaction scores. We found that correlated variables reflecting tumor biomarkers (CA125, CEA, CA15-3), clinicopathological features (tumor diameter, N stage, molecular subtype), and coagulation abnormalities (Fbg, FVIII, D-dimer) have important weights in predicting the recurrence of BC. This may result in more sustainable health for patients, thereby reducing the psychological, social and economic burden on society.

Our study has several limitations. First, the study population was relatively small. Although we evaluated 342 patients, 103 of whom were randomly included in the test set as an independent sample, a larger cohort is needed for future external validation of the accuracy of the prediction model. Second, although we initially evaluated the value of 25 available clinical features for predicting recurrence, we need more clinical information, such as gene mutations, to optimize these prediction models and provide a valuable basis for individualized treatment. Thus, future studies should be conducted to validate the feasibility of the proposed algorithm.

## Conclusion

This study described the application of clinical information and laboratory parameters-based ML in patients with BC recurrence, generating a AdaBoost algorithm model that reliably predicts the probability of BC recurrence. In our study, ML combined with the explainability method of SHAP makes the black box model of ML explainable, which is more suitable for the clinical scenario of predicting breast cancer recurrence. In addition, the addition of DCA highlights the clinical value of AdaBoost. We suggest the use of this approach as an auditable decision aid that contributes to patient healthcare and research.

### Electronic supplementary material

Below is the link to the electronic supplementary material.

**Supplementary material 1**: **Supplementary Table 1**. Clinical laboratory characteristics of breast cancer patients; **Supplementary Table 2**. The 3-fold cross-validation results of 11 machine learning models; **Supplementary Table 3**. Confusion matrix of 11 machine learning models

## Data Availability

The data generated in this study are available upon request from the corresponding author.
