# Supplementary material for: Machine learning-based models for the prediction of breast cancer recurrence risk
Source: BMC Med Inform Decis Mak. 2023 Nov 29;23:276. doi: 10.1186/s12911-023-02377-z (PMC10688055; doi:10.1186/s12911-023-02377-z)
Supplement: Supplementary file 1 — Supplementary material 1: Supplementary Table 1. Clinical laboratory characteristics of breast cancer patients; Supplementary Table 2. The 3-fold cross-validation results of 11 machine learning models; Supplementary Table 3. Confusion matrix of 11 machine learning models [file 12911_2023_2377_MOESM1_ESM.pdf]

Supplementary Materials

Machine Learning-Based Models for the Prediction of Breast Cancer Recurrence Risk

Duo Zuo[1,2,3,4,5,#], Lexin Yang[1,2,3,4,5,#], Yu Jin[1,6], Huan Qi[7], Yahui Liu[1,2,3,4,5], Li Ren[1,2,3,4,5,*]

Supplementary Table 1. Clinical laboratory characteristics of breast cancer patients.

Clinical laboratory characteristics	All patients	DR	No DR	*P*
RBC ($\times10^{12}$/L)	4.45 (0.48)	4.45 (0.51)	4.45 (0.43)	0.332
Hb (g/L)	133.00 (15.00)	132.00 (17.00)	134.00 (12.00)	0.073
WBC ($\times10^{9}$/L)	5.86 (2.23)	6.01 (2.51)	5.56 (1.72)	0.036
NEUT (%)	3.41 (1.82)	3.52 (1.93)	3.03 (1.27)	0.002
LYMPH (%)	1.86 (0.74)	1.83 (0.76)	1.94 (0.66)	0.071
PLT ($\times10^{9}$/L)	254.00 (77.00)	254.5 (78.00)	248.50 (76.00)	0.791
NLR (%)	1.77 (1.04)	1.89 (1.12)	1.56 (0.87)	0.000
PLR (%)	137.10 (62.25)	137.53 (64.48)	134.48 (56.86)	0.118
Fbg (g/L)	2.56 (0.78)	2.67 (0.77)	2.22 (0.52)	0.000
D-Dimer (ng/ml)	312.78 (292.50)	355.40 (369.20)	277.20 (163.41)	0.000
FVIII (%)	135.95 (56.22)	136.20 (65.75)	134.95 (45.63)	0.069
α2-AP (%)	110.80 (17.00)	111.62 (13.87)	105.25 (22.03)	0.000
TPSA (U/L)	47.54 (74.90)	54.25 (88.81)	39.60 (43.50)	0.058
CA15-3 (U/ml)	14.64 (18.18)	17.80 (24.47)	9.71 (7.50)	0.000
CA125 (U/ml)	12.07 (16.08)	15.83 (17.13)	1.47 (1.61)	0.000
CEA (μg/L)	3.59 (9.49)	2.39 (4.16)	11.49 (8.31)	0.000

For continuous variables, nonnormally distribution data were used median (interquartile intervals) and a Mann–Whitney test for comparison. CA125, carcinoma antigen 125; CEA: carcinoembryonic antigen; Fbg: fibrinogen; CA15-3, carcinoma antigen 15-3; FVIII, coagulation factor VIII; TPSA, tissue polypeptide-specific antigen; α2-AP, α2-antiplasmin; RBC, red blood cell; NEUT, neutrophils; PLR, platelet-to-lymphocyte ratio; WBC, white blood cell; PLT, platelet.

Supplementary Table 2. The 3-fold cross-validation results of 11 machine learning models.

Algorithms	Fold1	Fold2	Fold3	Average
AdaBoost	0.975	1.000	0.962	0.979
Decision Tree	0.950	0.950	0.962	0.954
GaussianNB	0.925	0.900	0.911	0.912
GBDT	0.963	1.000	0.962	0.975
LightGBM	0.988	0.988	0.962	0.979
LR	0.950	0.975	0.937	0.954
MLP	0.950	0.963	0.924	0.946
Random Forest	0.975	1.000	0.975	0.983
SVC	0.813	0.813	0.848	0.824
XGBoost	0.963	1.000	0.962	0.975
LDA	0.838	0.925	0.848	0.870

Supplementary Table 3. Confusion matrix of 11 machine learning models.

Algorithms	TP	FP	FN	TN
AdaBoost	18	2	1	82
Decision Tree	16	4	1	82
GaussianNB	16	4	8	75
GBDT	18	2	1	82
LightGBM	18	2	1	82
LR	19	1	3	80
MLP	18	2	3	80
Random Forest	18	2	0	83
SVC	9	11	3	80
XGBoost	18	2	1	82
LDA	13	7	5	78

TP, The number of samples that are correctly identified as positive; TN, The number of samples that are correctly diagnosed as negative; FP, The number of samples that incorrectly detected positive; FN, The number of samples that incorrectly detected negative.
